# Radiobiological shot noise explains Three Mile Island biodosimetry indicating nearly 1,000 mSv exposures

**DOI:** 10.1038/s41598-020-67826-5

**Published:** 2020-07-02

**Authors:** Aaron M. Datesman

**Affiliations:** 1 NASA Goddard Space Flight Center, Greenbelt, MD USA; 2 Department of Electrical and Computer Engineering, University of Virginia, Charlottesville, VA USA; 3 Washington, DC, USA

**Keywords:** Biological physics, Energy justice

## Abstract

The 1979 accident at the Three Mile Island nuclear power station in Pennsylvania released about 22 million Curies of xenon-133 into the environment. Because physical dosimetry indicated exposures to the nearby population of less than about 2 mSv, discernible impacts to the health of the exposed population are not generally believed to have resulted. However, there is contrary evidence, including especially the results of biodosimetry via cytogenetic analysis using the FISH method. This report examines the discrepancy between the results of physical dosimetry and biodosimetry, which among the small number of persons examined indicated exposures between 600 and 900 mSv. The paradox reveals a fundamental error in the health physics body of knowledge: the definition of the energy imparted to tissue, ε, fails to properly account for the temporal distribution of ionization products resulting from dilute contamination with an internally incorporated beta-emitting radionuclide. Application of a century-old result describing “shot noise” in an electronic system repairs the deficiency. The Xe-133 concentration in the tissue of those individuals exposed to the most intense portion of the radioactive plume released from the TMI facility is shown to have been on the order of 0.1 μCi/l, persisting for multiple hours. Shot noise reference doses in the range from 820 to 1,700 mSv follow, a result which is consistent with biodosimetric analysis. The finding should motivate a comprehensive re-evaluation of the conventional understanding of the 1979 accident at the Three Mile Island nuclear power station, especially regarding its impact upon the population of the surrounding area.

## Introduction

The 1979 accident at the Three Mile Island (TMI) nuclear power station in Pennsylvania released a large quantity of the radioactive noble gas xenon-133 into the surrounding environment. Although it is well-established that gamma ray exposures to the affected population were comparable to or smaller than the annual dose due to background radiation (around 1 mSv), the topic of health effects relating to the accident has always been controversial. The Report of the President’s Commission on the Accident at Three Mile Island (known as the Kemeny Commission Report, after its chairman) asserted that any health or medical impacts affecting the population living within twenty miles of the accident site were due to mental distress^1^. On the basis of what is known about the accident and the nature of the exposure suffered by those nearby, it is therefore not conventionally believed that any discernible impact to human health caused by exposure to ionizing radiation has been observed^2–4^.

However, contrary evidence does exist, and ought not to be summarily dismissed. For instance, contemporaneous accounts from hundreds of local residents describe symptoms consistent with significant exposure to ionizing radiation, including erythema, hair loss, nausea, and vomiting^5^. Researchers later correlated more than a dozen verified reports of medical impacts to simultaneous meteorological conditions at the TMI facility^6^, at least suggesting the presence of the radioactive plume at the location of the individuals making the reports. Because the persons affected in some cases were not aware that a radiological release from the TMI facility had occurred following an accident, and in most or all cases may not have been knowledgeable regarding the medical impacts of radiation poisoning, a diagnosis of mental distress as an explanation for the acute effects observed is difficult to accept.

Furthermore, large-scale epidemiological investigations have uncovered health decrements including breast and lung cancer, heart disease, and early mortality among the exposed population within ten miles^7^ and five miles^8–10^ of the TMI facility. Because the doses suffered by the exposed population were small and because the discernible health impacts were not those expected for the nature of the exposure, among other reasons, most investigators have been unwilling to interpret these epidemiological results as convincing evidence relating the observed health impacts to exposure to ionizing radiation. Due in part to their results showing a clear dose response for lung cancer, however, a group of researchers from the University of North Carolina at Chapel Hill contended that the emissions from the accident should be considered causative for the observed excess incidence of cancer in the surrounding ten-mile area^11^. In order to justify their conclusion, the UNC researchers hypothesized that the doses to the affected population may have been much higher than generally accepted. There is little corroborating evidence for the claim. The Kemeny Commission, for instance, concluded that the greatest exposure to any individual due to the accident was only about 0.7 mSv.

The present article engages specifically with one piece of evidence cited by the UNC team supporting their view: the results of the cytogenetic analysis of 29 individuals, living near TMI at the time of the accident, who reported symptoms consistent with radiation poisoning contemporaneous with the accident^12^. The analysis produced dose estimates in the range of 600–900 mSv, orders of magnitude larger than the gamma ray doses estimated by the Kemeny Commission, the Nuclear Regulatory Commission^13,14^, experienced nuclear industry consultants^15,16^, or the Three Mile Island Public Health Fund^17^. While the results of the cytogenetic analysis have been published in the open scientific literature and are freely available, the existence of this information does not appear to be widely known.

While the dose estimate based upon cytogenetic analysis is sufficient to explain contemporaneous reports of acute effects, then, it contradicts firmly established estimates of the gamma dose to affected individuals. Because those gamma ray dose estimates are anchored to actual, physical measurements taken by dosimeter at the time of the accident, the cytogenetic results are difficult to explain. Although the conflict seems irreconcilable, in fact its existence illuminates a fundamental oversight in the health physics body of knowledge^18^. While the phenomenon of shot noise^19^ deriving from the discrete nature of electrical charge has long been known to apply to biological systems at least in the context of nerve-muscle junctions^20^ and membrane conduction^21^, its application to radiobiology has up until the present time been neglected.

It will be shown (contrary to the assertions of authorities) that the gamma ray doses suffered by those in the path of the Xe-133 plume were far from the most significant exposures that occurred. The results of cytogenetic analysis are instead consistent with the effects of internal exposure to beta radiation. Correcting a fundamental oversight in the health physics body of knowledge—relating to shot noise in the context of radiobiology—resolves the apparent paradox. The finding should motivate a comprehensive re-evaluation of the conventional understanding of the 1979 accident at the Three Mile Island nuclear power station, especially regarding its impact upon the population of the surrounding area.

## Materials and methods

### Results of cytogenetic analysis

The microscopic study of chromosomes (termed cytogenetics) began in the 1930s and has been well-established for many decades. The field evolved jointly (frequently collaboratively) with the study of x-ray mutagenesis. Important early workers in these two fields included the American Nobel prizewinners Barbara McClintock^22^ and H.J. Muller^23^. Due to their discoveries, along with the efforts of countless others, it has long been established that a) mutations due to ionizing radiation can be stably inherited across multiple generations, and b) the mutation rate increases linearly with dose^24^. The scientific heritage embodied by these findings was the genesis of the idea, first suggested in the early 1960’s, that chromosomal aberrations might serve as a kind of retrospective biological dosimeter^25^. Practical cytogenetic dosimetry in humans was well-established by the late 1960s^26^. The International Atomic Energy Agency (IAEA) has maintained a program in biodosimetry for several decades and recommends that knowledge in this area should be employed to guide response in case of a radiation emergency^27^.

Because in fact not all chromosomal anomalies are stably inherited across cellular generations, the general picture is more complex if dosimetry is contemplated many years or decades after exposure. In fact, the dicentric aberrations from peripheral blood lymphocytes typically employed for biodosimetry via "conventional" cytogenetic analysis are cleared from the body on an uncertain time scale of about three years or more^28^. Chromosomal aberrations of this kind are termed “unstable.” Since the mid-1990s, however, it has been possible instead to analyze for stable chromosome aberrations using the FISH (Fluorescent In-Situ Hybridization) method^29^. Biodosimetry using the FISH method is today well-established and has been employed to examine exposed populations including the survivors of the atomic bombings at Hiroshima and Nagasaki^30,31^, radiation workers at Sellafield^32^, atomic test veterans^33^, and even astronauts on the International Space Station^34^. It is conventionally believed that population doses not lower than 100 mSv may be determined retrospectively using conventional cytogenetic methods^35^, while the threshold for the minimum detectable dose using the FISH method is 200–300 mSv^36–40^.

Since the sensitivity threshold for cytogenetic analysis of 100 mSv far exceeds the exposures understood to have occurred in the area surrounding the Three Mile Island nuclear power station in March–April 1979 (not greater than 2 mSv), state and national authorities of the United States appear not to have performed cytogenetic testing of any affected persons in an attempt to quantify the exposures due to the accident. Nevertheless, in the course of litigation such testing was performed on a relatively small number of persons, aged 16 to about 60 years^12^, in 1994–1995 (fifteen years after the accident). The cytogenetic analysis was performed by experienced researchers from the Russian Academy of Sciences, who had previously applied these same methods to examine populations exposed by the Chernobyl accident^41,42^, as well as releases from the Mayak plutonium production facility and fallout due to Soviet nuclear testing^43^. The results relevant to the Three Mile Island accident, published in an academic format in a conference proceedings in English, as well as in a Russian-language journal^44^, are given in Table 1.

**Table 1:** Summary of the results of cytogenetic testing, from^43^.

		Conventional			FISH			
		#	Cells	C_dr_ (/1,000)	#	Cells	F_p_ (/100)	F_g_ (/100)
Altai-LL (alive 1949)	Exposed	84	22,195	1.9 ± 0.3	14	7,026	0.41 ± 0.08	1.29 ± 0.11
	Exposed				8	4,271	0.58 ± 0.12	1.82 ± 0.17
	Control	30	7,831	0.3 ± 0.2	12	13,586	0.10 ± 0.03	0.32 ± 0.05
TMI	Exposed	29	14,854	2.0 ± 0.4				
	Exposed	6	3,024	4.6 ± 1.2	6	3,468	0.49 ± 0.12	1.55 ± 0.21
	Control	82	26,849	0.2 ± 0.1				

The table describes the number of persons examined, the number of cells scored, and the rate of unstable dicentric and ring chromosomal aberrations, as well as the frequency of stable translocations and the genomic translocation frequency as determined by the method of Fluorescence In-Situ Hybridization (FISH). FISH analysis of the population of Laptev Log in the Altai Mountains (Altai-LL) revealed that the principal exposure occurred in 1949, subsequent to the Soviet Union’s first nuclear test. The rate of stable translocations found among six persons living near Three Mile Island at the time of the 1979 accident indicates exposure nearly as great as that found among villagers exposed to bomb fallout in the Soviet Union.

Table 1 summarizes the results of both conventional cytogenetic analysis (examining both dicentric and centric ring chromosomal aberrations) and FISH analysis (examining symmetrical translocations) for the examined populations in two locations: residents of the village of Laptev Log in the Altai Mountains (“Altai-LL”), as well as residents of Pennsylvania living near Three Mile Island at the time of the 1979 accident (“TMI”). The Laptev Log exposures occurred subsequent to the Soviet Union’s first test of an atomic weapon in 1949, which irradiated a large inhabited area in southern Siberia northeast of the test site in Kazakhstan. Experimental measurements and mathematical simulation revealed that the residents of Laptev Log received exposures of about 970 mSv as a result of the 1949 nuclear test.

The data labeled “Conventional” in Table 1 clearly indicate greatly elevated rates (by a factor of six to ten or more) of unstable chromosomal aberrations in the exposed populations compared to reference levels. The measured rates of chromosomal aberrations in the control populations were similar in both locations. The conventional cytogenetic results are strong evidence that significant exposures occurred among the population affected by the accident at Three Mile Island. However, because the types of chromosomal aberrations described are unstable with elapsed time, no retrospective dosimetric evaluation for either population can be supported on the basis of the cytogenetic testing results. For this reason, six persons from among the twenty-nine individuals from Pennsylvania for whom conventional cytogenetic testing was performed were selected for further evaluation using the FISH method.

The following expression relates the frequency of stable translocations *f*_*trans*_ to the absorbed dose *D*, measured in Grays^36,45^:

1 $${f}_{trans}={10}^{-3}\left\{0.96+9.5D+14.5{D}^{2}\right\}.$$

Applying Eq. (1), which assumes the dose to be prompt/acute in character, the measured frequency of stable translocations among the residents of Laptev Log who were alive in 1949 indicates an exposure of approximately 370 mGy. (Residents of Laptev Log born after 1949, who were not exposed to fallout from the Soviet Union’s first nuclear test, showed a rate of stable translocations not significantly different from the control.) The dosimetry is complex, however, since the exposure was due to environmental contamination, difficult to characterize, and chronic or prolonged in character. Because acute doses are more efficient at producing chromosomal damage^46^, an upward adjustment by a factor of two to three is justified^44^. In this manner one arrives at an exposure among the population of Laptev Log of approximately 1,000 mSv, which agrees with the result obtained from physical dosimetry and mathematical modeling.

A rate of stable translocations found among the TMI population similar to that found for the residents of Laptev Log (0.49 versus 0.58 translocations per 100 cells) implies a biodosimetric result of a similar magnitude. Equation (1) yields a value for *D* = 300 mGy, which if one applies the adjustment for non-acute exposures produces a dose estimate for those affected in the range of 600–900 mSv. While the result possesses a solid foundation and is consequently well-justified, it is important to acknowledge several significant caveats. First, there is no documentary evidence that the results were adjusted for age, which would be a requirement in the present day^47^. Second, there was no distinct control group for TMI FISH analysis. The researchers used the population from Laptev Log for this purpose. Third, since the duration of the TMI exposures is not really clear, it is uncertain that the additional factor of 2–3 × for protracted exposures is justified. Fourth, and finally, it is proper to ask whether testimony submitted in the course of a legal dispute is acceptable as a foundation for a scientific investigation.

The concern about age adjustment is entirely valid and would be a valuable area for follow-up expert review. Regarding the second concern, the finding from conventional cytogenetic testing that the rates of dicentric and ring chromosomal aberrations were similar between the control groups at both locations (each consisting of more than eighty persons) is reassuring. As to the third point, it will be shown that the duration of the TMI exposures exceeded twenty-four hours. The exposure therefore certainly cannot be described as acute in character. Finally, the researchers who performed the work were well-credentialed in the Soviet Union and possessed decades of relevant experience. While it is naturally vital to maintain a healthy level of skepticism on any controversial topic, there is no justification for failing to at least consider the results of cytogenetic testing in this situation.

### Nature and magnitude of the Xe-133 exposure

Estimates of the release rates provided by the Nuclear Regulatory Commission correspond to an overall source term due to the TMI accident of about 7 million Curies (MCi) Xe-133^48^. According to the report of the Kemeny Commission, noble gas emissions due to the accident were 13 MCi or less, with whole-body gamma doses to the local population not greater than 0.7 mSv. Researchers working for the Three Mile Island Public Health Fund several years later (henceforth referred to by their initials, BDC) concluded that the likeliest value of the source term was 22 MCi, with doses to individuals from the Xe-133 release as large as about 2 mSv^17^.

**Figure 1:**
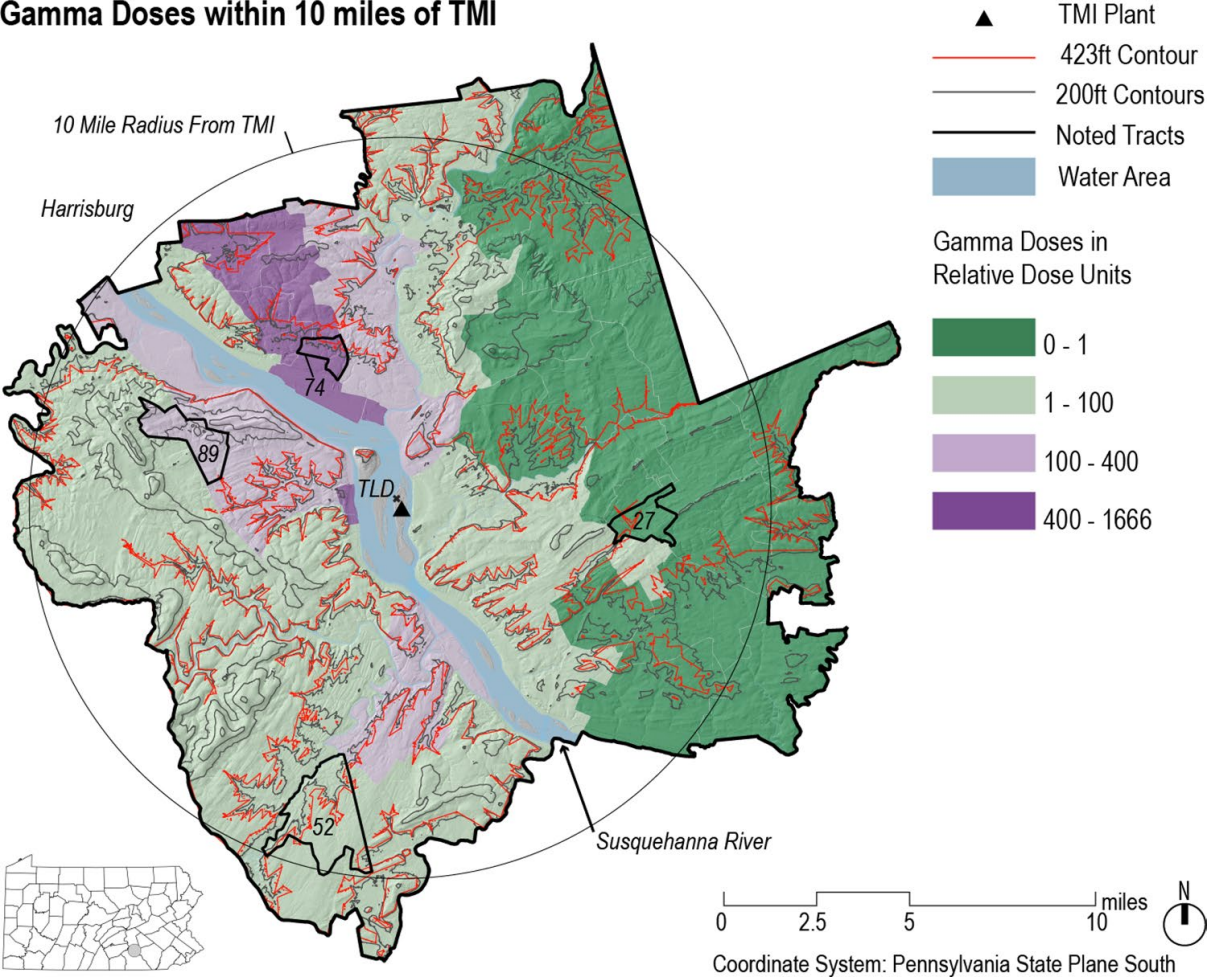
Gamma dose estimates by BDC for the region within ten miles of Three Mile Island, reduced to four categories of exposure for ease of visualization. The northwesterly path of the most intense releases is clear. In the base case scenario, the peak value of 1666 relative dose units corresponds to a gamma dose estimate of 1.1 mSv. The 423-foot contour corresponds to the elevation of the release point. The TLD at the indicated location recorded a gamma dose of 10.26 mSv. The map was created using the QGIS 2.14 software package (https://qgis.org).

BDC based their analysis upon meteorological data, available in 15-min increments, along with the readings of twenty thermoluminescent dosimeters (TLDs) surrounding the TMI facility. Only four of the dosimeters registered exposures greater than 1 mSv. The wind direction was measured with an accuracy of five degrees. As shown in Fig. 1, BDC determined that the most significant exposures occurred northwest of the reactor, coinciding with the wind direction during the hours of peak Xe-133 emission. Therefore the seven tracts northwest of TMI with doses between 400 and 1,600 relative dose units are referred to as “downwind”.

Because information about the effluent temperature was not captured during the accident at TMI-2, the base case scenario cannot be regarded as definitive. Since a plume with greater rise must contain more activity in order to generate the ground level doses measured by TLD, there is very significant uncertainty related to plume rise. BDC dealt with this uncertainty in two ways. Firstly, the authors considered a range of values for the plume rise, between zero and high thermal buoyancy. For temperature differences of 0, 10, and 100 °C degrees, BDC found maximum gamma doses at any receptor equal to 1.0, 2.1, and 3.8 mSv, respectively, with total releases of 8.6, 22.1, and 45.4 MCi. The base case (most likely) scenario was judged to be the modest temperature difference of 10 °C. Secondly, BDC defined their results using a relative scale, measuring the gamma dose between zero and 1666 relative dose units. Using a relative scale to measure exposure preserved the ability to compare outcomes between tracts (supporting epidemiological investigation) without specifying the maximum dose in physical units.

Consideration of the thermal rise uncertainty emphasizes the nature of the BDC analysis, which is the outcome of a model anchored to measurements. The dose estimates across more than sixty tracts illustrated in Fig. 1 do not themselves directly represent measurements of gamma dose. If the chosen model assumptions were not well-justified or did not reflect the actual meteorological conditions existing near TMI at that time, then the results of the BDC model might not be strictly correct. The concern is legitimate because direct measurements of the Xe-133 plume made by helicopter at the time of the accident (although hours after the period of greatest release) revealed it not to conform to a classical Gaussian plume shape, even exhibiting a tendency to "puddle" under conditions of light wind^49^. Nevertheless, for purposes of discussion and clarity this report assumes the BDC base case analysis to be correct.

The analysis performed by BDC divided the hours following the accident into seven distinct time segments, covering 38 h in total. The release rate within each segment was assumed to be constant. BDC utilized the available meteorological data with a variable-trajectory puff model to calculate the gamma dose to each receptor via a point-kernel method of integration over the extent of each puff at a given time. The spatial extent of each puff was characterized using the Pasquill–Gifford dispersion coefficients, consistent with experimental results for dispersion of a continuous-release plume^50^. The results are summarized in Table 2. BDC concluded that most of the Xe-133 release occurred during two distinct intervals (Interval 3 and Interval 4) beginning at 14:00 h on 28 March 1979. 80% of the total emissions were released during Interval 3 and Interval 4.

**Table 2:** Time intervals and base case release rates as determined by BDC^17^.

Interval	Times of day (28–29 March)	Duration (h)	Release Rate (MCi/hr)	Activity (MCi)	Wind direction	${{\varvec{D}}}_{{\varvec{\gamma}}}$(mSv)	[Xe-133] (μCi/l )
1	04:00–11:45	7.75	0.067	0.52	Shifting		
2	11:45–14:00	2.25	0.287	0.65	S		
3			1.040	9.36			
3a	14:00–17:00	3.00			SSW		
3b	17:00–23:00	6.00			SSE	0.9	0.55
4	23:00–00:15	1.25	6.600	8.25	SSE	1.2	3.5
5	00:15–01:30	1.25	0.038	0.048	S		
6	01:30–07:30	6.00	0.398	2.39	Shifting		
7	07:30–17:15	9.75	0.106	1.03	Shifting		
			TOTAL	22.3		2.1	

The wind blew steadily to the northwest with Class E stability conditions beginning at 17:00 h on 28 March 1979, throughout all of Interval 3b and Interval 4. The gamma dose and ground-level Xe-133 concentration are calculated using the point-kernel method, assuming a plume rise of 50 m in accordance with the base case scenario.

Published data indicate that the wind measured at TMI blew steadily to the northwest, with class E stability conditions, beginning at 17:00 h March 28, partway through Interval 3. The shift in wind direction motivates the division of Interval 3 into two segments, Interval 3a and Interval 3b. Because the wind direction was consistent to the northwest during all of Interval 3b and Interval 4, the exposures received in downwind tracts accrued almost solely during these hours, when the recorded wind speed lay between 3.2 and 4.5 m/s. The average wind speed was u = 3.9 m/s^51^. The data describing wind speed and direction utilized in this report are resolved to one hour and 22.5 degrees, respectively, which is inferior to the meteorological data employed by BDC.

For the base case scenario with a 10 C° temperature difference, BDC found the greatest dose encountered in any study tract to occur in Tract 74, an area located approximately 7.37 km northwest of the TMI-2 vent stack. The tract is labeled in Fig. 1. The Tract 74 gamma dose of 1.1 mSv was averaged spatially over not fewer than ten receptors, with the greatest dose calculated at a single receptor of ${D}_{\gamma }$ = 2.1 mSv. The difference between the maximum and average doses implies a narrow plume and very steady wind conditions (that is, constant puff trajectories). Using the standard expression for a Gaussian plume, along with the PG dispersion parameters, the Xe-133 release rates calculated by BDC, and an assumed 50 m plume rise, a point-kernel calculation gives a ground-level gamma dose of 0.90 mSv during Interval 3b, followed by an additional 1.20 mSv during Interval 4. The combined gamma dose of 2.1 mSv corresponds precisely to the BDC result. The calculated Xe-133 concentration at the receptor is *χ* = 0.55 μCi/l during Interval 3b, increasing to 3.5 μCi/l during Interval 4. For reference, the limit set by the Nuclear Regulatory Commission for occupational exposures to airborne Xe-133 is 0.1 μCi/l ^52^.

### The kinetics of inhaled xenon gas in the human body

The half-life of Xenon-133 is 5.25 days. The radioisotope decays with a total energy of 427.4 kilo-electron Volts (keV). In its principal decay path, this energy is divided between an 81 keV gamma ray and the kinetic energy of a beta particle, up to a maximum of *Q* = 346.4 keV. The average beta particle kinetic energy *E*_*av*_ = 100.5 keV^53^. Because the mean free path of a 100 keV beta particle in air (0.13 m)^54^ is far less than the mean free path of an 81 keV gamma ray (50 m)^55^, the external gamma ray dose dominates other contributions to the total dose received due to immersion within a dilute cloud of Xe-133. According to the conventional understanding, it follows that the beta dose may for the most part be neglected. Anticipating the shot noise result of the next section, however, it is worthwhile to examine the situation more deeply.

A person immersed in a cloud of Xe-133 will suffer internal exposure via the inhalation pathway in addition to external gamma-ray and beta-ray exposures. For example, the concentration of Xe-133 in the lungs will be equal to the ambient concentration in air. The internal beta-ray exposure is not confined to tissue in the lung and respiratory pathway, however. Based upon fundamental considerations, one expects that gaseous xenon will be absorbed into the blood from the lungs^56,57^. Furthermore, because the hemoglobin molecule possesses a significant affinity for xenon^58^, circulating blood carries dissolved Xe-133 throughout the entire volume of the body, which observation explains the utility of gaseous xenon as an anesthetic agent^59,60^. The uniform distribution of Xe-133 via the circulatory system creates a uniform whole-body beta dose to tissue due to internal incorporation of the radionuclide. Because of shot noise, the biological effect of this exposure can be very great.

Investigations examining the uptake of Xe-133 in human subjects were performed by researchers in the Soviet Union using a hermetic exposure chamber^61^. The researchers observed that the biological half-life of Xe-133 in lungs and blood is only 30 s, indicating free exchange between the lungs and the circulatory system. The distribution factor in human blood (alternatively known both as the blood-gas partition coefficient, and as the Ostwald coefficient, *λ*) was measured to be *λ* = 0.17 ml/g (averaged among four test subjects) after nine hours of exposure. The value is somewhat higher than the value of *λ* = 0.14 ml/g generally accepted in the anesthesiology community. There is additional evidence, including recent experimental results, that the value might be as low as *λ* = 0.115 ml/g^62–64^.

While the blood-gas exchange describes how Xe-133 enters the human body, the residence time and distribution of the radionuclide in tissue are more complex. The noble gas is soluble in tissue—in fat especially—and is observed to both accumulate and dissipate following exponential curves with time constants as large as several hours. Full saturation is observed only after an exposure duration exceeding about twenty hours. The results published by Turkin and Moskalev, presented in Table 3, summarize the relevant findings^61^. It is notable that the distribution factor in fat tissue is nearly ten times larger than the blood-gas partition coefficient.

Using the constant values of the ambient Xe-133 concentration during Interval 3b and Interval 4 from Table 2 and the half-lives of Xe-133 dissolved in tissue from Table 3, it is possible to create a time profile of the concentration of this noble gas in human tissue due to inhalation following release from the TMI facility. For example, for absorption toward saturation the concentration in tissue is given by the expression.

**Table 3:** Parameters describing the kinetics of Xe-133 in the human body, as determined by researchers in the Soviet Union^61^.

	*λ* (ml/g)	_*τabs*_	_*τrel*_
Fat tissue	1.4	5 h	6.3 h
Muscle and other tissue	0.13	0.4 h	0.7 h
Blood	0.17	30 s	30 s
Lungs	2	30 s	30 s

*τ*_*abs*_ and *τ*_*rel*_ represent the half-lives for absorption by and release of Xe-133 from tissue, respectively.

2 $$c\left(t\right)={c}_{o}+(\lambda \rho \chi -{c}_{o})\left[1-exp(-t\cdot ln2/{\tau }_{abs})\right].$$

For the release from tissue after dissipation of the external plume, the concentration in tissue instead has the form.

3 $$c\left(t\right)={c}_{o}exp(-t\cdot ln2/{\tau }_{rel}).$$

In Eqs. (2) and (3), *c* represents the concentration of Xe-133 in tissue (in units of μCi/l), *c*_*o*_ is the concentration *c* at time *t* = 0, *ρ* is the density of water, and the term *ln(2)* arises because the time constants are defined as half-lives. The expressions of Eqs. (2) and (3) describe responses to stepwise changes in the ambient concentration either upward, from an initial concentration *c*_*o*_ possibly different from zero, or downward, to zero concentration.

**Figure 2:**
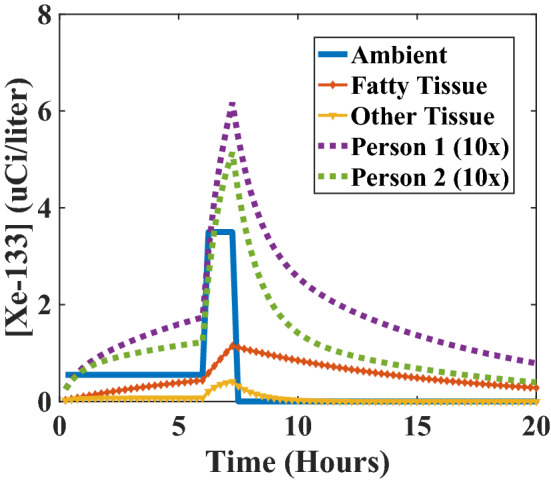
Concentrations of xenon-133 in the atmosphere (solid line), in fatty and other tissue (marked lines), and averaged according to Eq. (10) for Person 1 and Person 2 (dashed lines, magnified by 10 × for clarity).

The time profile of the Xe-133 concentration in tissue is shown in Fig. 2. The figure demonstrates that the tissue of persons in Tract 74 during the evening hours of 28 March 1979 was contaminated with concentrations of Xe-133 on the order of 0.1 μCi/l. The result was a uniform internal exposure to tissue due to energetic beta particles, with a range in tissue of about 1 millimeter^54,65^. The exposure persisted for more than forty hours.

Realistically, because the intervals of greatest release included evening and late evening hours when most persons affected were indoors, and because overnight temperatures were cold (between 30 and 40 degrees Fahrenheit) in Middletown, PA, on 28 March 1979, shielding may have had some impact and should be considered. Under cold weather conditions in residential buildings of that era in Pennsylvania, it is reasonable to assume neutral pressurization resulting in infiltration at a rate of one air change per hour (1 ACH)^66^. A simple description of air exchange using a single time constant to characterize both infiltration and exfiltration yields a peak indoor concentration of around 2.5 μCi/l, about 30% lower than the ambient value outside. However, because infiltrated air does not dissipate immediately when the external plume is removed, an increased duration of exposure is found to compensate for the lower indoor peak concentration. Because the net result is found to be little different from the case with no shielding, only the case of exposure to the outdoor ambient concentration is considered further.

The next section describes the shot noise result for the time-averaged power dissipated in tissue due to radioactive decay. Due to shot noise, it is dramatically incorrect to neglect the effect of the internal exposure to Xe-133 just derived.

### The biological effect of an internally incorporated beta-emitting radionuclide

**Figure 3:**
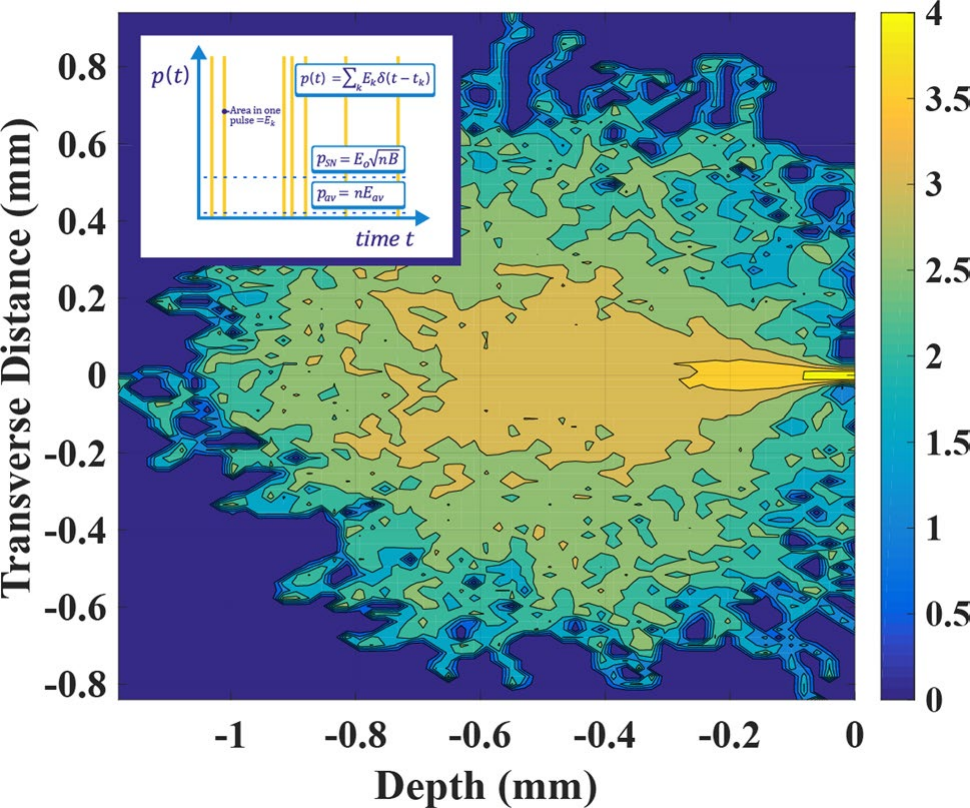
Illustration of the beta particle interaction volume in cross-section for *Q* = 364.4 keV, obtained by Monte Carlo modeling with two million individual interactions. The volume is determined by rotation of a two-dimensional slice in the transverse direction around the central axis. The image was created using the 2016 version of MATLAB (www.matlab.com). The color scheme is logarithmic. (inset) Schematic describing the pulse train *p*(*t*) of Eq. (4), illustrating the difference between the instantaneous power, the average power, and the shot noise power.

As a model of living tissue, consider a quantity of water (denoted sample A) in which Xe-133 is dilutely incorporated. The chemically reactive free radicals generated in sample A by radiolysis following beta particle emission possess a short lifetime on the order of 1 ns^67–69^ and are confined within a small interaction volume V delimited by the beta particle range. The results of Monte Carlo modeling^70,71^ for electrons with the maximum kinetic energy *Q* = 364.4 keV, illustrated in Fig. 3, indicate that the interaction volume possesses the shape of a teardrop^72^, with a volume of about *V* = 1.6 mm^3^.

As an example, assume the Xe-133 concentration in sample A to be equal to the ambient atmospheric concentration during Interval 4, *c* = 3.5 μCi/l. In this case, on average one instance of beta decay occurs in a given interaction volume only every 4.8 s. Compared to the time scale set by the rate of the chemical reaction, decays are very infrequent, and a state of chemical equilibrium exists at most instants of time. However, when a decay does occur, the instantaneous power dissipation and the degree of chemical disequilibrium are very large relative to their average values. The instantaneous power dissipation *p*(*t*) in the volume *V* is accurately described using a pulse train:

4 $$p\left(t\right)=\sum_{k}{E}_{k}\delta (t-{t}_{k}).$$

In Eq. (4), *δ* represents the Dirac delta function, with infinitesimal width and unit area, while *E*_*k*_ represents the kinetic energy of the kth beta particle emitted at random time *t*_*k*_*.* The time-averaged power dissipation $\bar{p}$ is given by the equation^18,19^.

5 $${\bar{p}}^{2}={E}_{o}^{2}nB+{E}_{av}^{2}{n}^{2}.$$

In Eq. (5), *E*_*o*_ and *E*_*av*_ represent the expected value and the average value of the beta particle kinetic energy spectrum, respectively, the pulse emission rate *n* = *cV*, and *B* represents the bandwidth expressing a limit to the speed of the chemical reaction that dissipates power in solution and is responsible for biological injury. The value of *B* = 1.59 × 10^8^ s^−1^ corresponds to the hydroxyl radical lifetime of 1 ns.

Recognizing that the average power is given by the expression ${p}_{av}={\int }_{0}^{T}p\left(t\right)dt/T$, it follows that the second term on the right-hand side of Eq. (5) represents the average dissipated power: ${p}_{av}={nE}_{av}$. The first term, on the other hand, exists to account for the high level of instantaneous dissipated power associated with a single, individual pulse, or “shot”. The excess contribution due to this term is denoted the shot noise dissipated power,

6 $${p}_{SN}={E}_{o}\sqrt{nB}.$$

Equation (5) for the time-averaged dissipated power may therefore be easily understood as the sum in quadrature of two independent contributions: ${\bar{p}}^{2}={p}_{SN}^{2}+{p}_{av}^{2}$. In most practical circumstances *n* <  < *B*, so that the shot noise dissipated power dominates the average power by orders of magnitude. In short, the shot noise contribution to the time-averaged dissipated power may not be neglected and ought not to be overlooked.

The formalism of microdosimetry offers a convenient means by which to determine the reference dose to the model tissue of sample A. According to this framework, the energy *ε* imparted to a volume of interest is defined as the sum^73^.

7 $$\varepsilon ={\sum }_{i}^{N}{\varepsilon }_{i}.$$

In Eq. (7), *ε*_*i*_ is a stochastic quantity representing the energy deposited by a single ionization event or interaction. The random character of the energy deposits arises because the approach focuses on small interaction volumes (about one micron or less in size) corresponding to the size of biological targets of interest. The fluctuations considered are spatial in nature since ionization events occur in certain discrete locations—and are even clustered together in some instances—but are absent in others. The reference or equivalent dose is defined as the ratio of the mean energy $d\bar{\varepsilon }$ imparted to the volume of interest to the mass of that volume, *dm*.

Considering that the pulse train expression of Eq. (3) describes fluctuations of a temporal, rather than spatial, character, it is obvious that Eq. (7) is not perfectly general: the expression in no way accounts for the temporal distribution of ionization events. The approach is not suitable to describe chemical effects arising from the instantaneous concentration of free radicals, the class of so-called “indirect effects”. A more general expression accounting for the temporal distribution of ionization events within the well-defined interaction volume *V* is.

8 $$\varepsilon ={\int }_{0}^{T}p\left(t\right)dt.$$

In Eq. (8), *p(t)* refers to the pulse train expression of Eq. (4), while *T* represents the duration of exposure. The expression is merely a clear statement of the basic relationship between energy and instantaneous dissipated power.

It follows from Eq. (8) that the mean energy imparted to the interaction volume is $d\bar{\varepsilon }=T\bar{p}$, with $\bar{p}$ given by the expression for the time-averaged dissipated power, Eq. (5). In order to contextualize the foregoing, now consider a second small volume of water, sample B, irradiated with x-rays in order to replicate the power dissipation within and the chemical state of sample A. The x-ray dose to sample B is simply the reference dose to sample A, ${D}_{ref}=d\bar{\varepsilon }/dm$. The result is.

9 $${D}_{ref}=\frac{{E}_{o}T}{\rho V}\sqrt{cVB}.$$

Because *n* <  < *B* in virtually any imaginable practical situation, one expects the reference dose to sample B to be orders of magnitude larger than the beta dose to sample A responsible for the chemical effect.

Since the reference dose represents a notional exposure to x-rays, Eq. (9) is compatible with the Linear No Threshold (LNT) framework describing x-ray carcinogenesis^74^. The reference doses *D*_*ref*_ in successive time short time intervals *dt* with constant concentrations *c(t)* may therefore be straightforwardly summed. It follows that it is a simple matter of integration to determine the reference dose when the concentration *c* is changing in time, as illustrated for instance in Fig. 2.

## Results

Fundamentally, this report addresses a single question, motivated by the nature and magnitude of the exposures believed to have occurred immediately following the accident at Three Mile Island. What is the single best value expressing the magnitude of an x-ray reference dose generating a degree of chemical disequilibrium equivalent to that resulting from the non-uniform internal incorporation of radioxenon at concentrations of about 0.1 μCi/l? Because the human body is structurally complex across many scales of length and because xenon dissolves preferentially in fatty tissue, it is not immediately obvious how properly to apply the result of Eq. (9) to address the question. Nevertheless, insight can be obtained from the examination of simple models. For instance, since every human body consists of some combination of fatty and non-fatty tissues, consideration of each sort of tissue on its own describes two limiting cases. Applying Eq. (9) to the time series concentrations illustrated in Fig. 2 and integrating numerically, the reference doses to fatty and non-fatty (“other”) tissues are found to be *D*_*fat*_ = 2,900 mSv and *D*_*other*_ = 470 mSv, respectively. The limiting values for non-fatty and fatty tissues accord with the results of cytogenetic analysis in the range of 600–900 mGy.

Two simple models may additionally be suggested. If fatty and non-fatty tissues are intermingled on length scales of less than about one millimeter (the beta particle range), for instance, then one might consider modeling the inhomogeneous distribution of Xe-133 throughout the human body with an average concentration weighted by the percentage of body fat,

10 $${c}_{avg}=f{c}_{fat}+(1-f){c}_{other}.$$

In Eq. (10), *f* represents the percentage of body fat, *c*_*fat*_ the concentration of Xe-133 in fatty tissue, and *c*_*other*_ the concentration of Xe-133 in muscle and other tissue. The average Xe-133 concentration *c*_*avg*_ is then utilized in Eq. (9) to calculate the reference dose.

On the other hand, if fatty and non-fatty tissues are distinct on length scales of one millimeter and greater—so that the concentration of Xe-133 can be sensibly defined as spatially inhomogeneous—it is nevertheless true that the reference dose of x-rays refers to a spatially homogeneous whole-body exposure. In this case, one might consider instead weighting by the reference doses to each kind of tissue:

11 $${D}_{ref}=f{D}_{fat}+(1-f){D}_{other}.$$

In Eq. (11), *D*_*fat*_ and *D*_*other*_ each refer to the reference dose result of Eq. (9) using the individual Xe-133 concentrations in fatty and non-fatty tissue, respectively. The results of calculations for two archetypical persons, using both approaches, are given in Table 4. The results lie between 820 and 1,700 mSv, which corresponds reasonably well (perhaps higher by a factor of less than two) to the results of cytogenetic testing. A comprehensive attempt at error analysis is not justified because, among other factors, the author is not aware whether any of the individuals who underwent cytogenetic testing were in fact in or near Tract 74 at the time of the accident. Nevertheless, the demonstrated agreement is sufficient to justify further careful investigation.

**Table 4:** Whole body reference doses using different weightings for fatty and non-fatty tissue, for two persons with different percentages of body fat.

Person	% Body fat	Reference dose D_ref_ (mSv)	
		Weighted by concentration	Weighted by dose (whole body)
1	28	1,700	1,200
2	14	1,300	820

The beta doses to Person 1 and Person 2 are 9 μGy and 6 μGy, respectively, three orders of magnitude less than the gamma ray dose and nearly six orders of magnitude less than the reference dose representing the true biological impact.

## Discussion

The work described in this report about the 1979 incident at the Three Mile Island nuclear power plant in Pennsylvania is motivated by a paradox: the published results of biodosimetry and of physical dosimetry (coupled with the modeling of atmospheric dispersion) relating to the accident disagree by a factor of around 1,000. It has been shown that the paradox is neatly resolved if one separates the causes of the biological and physical dosimetric results while simultaneously addressing a serious oversight in the field of microdosimetry. Regarding the first point, while the physical dosimetry supports assessment of the degree of external exposure to gamma rays (around 2 mSv), this report has argued that the biodosimetric result arises from internal exposure to the beta-emitting radionuclide Xe-133. Regarding the second point, the conventional expression for the energy imparted to tissue, ε, does not consider the temporal character of energy deposition and therefore cannot account properly for the nature of the chemical insult to tissue resulting from exposure to an internally incorporated beta-emitting radionuclide. The general expression of Eq. (8)—which reduces to the conventional expression of Eq. (7) for *n* >  > *B*—must be utilized instead. The surprising numerical results presented therefore essentially derive straightforwardly from a basic application of the calculus, which should not be in the least controversial.

Since it is widely and commonly believed that no one was harmed by the Three Mile Island accident, certainly it will be found controversial to assert that exposures equivalent to nearly 1,000 mSv did occur and have been legitimately verified in the population living near the TMI facility at the time of the accident using biodosimetry. The assertion is provocative and seems, superficially at least, to be highly unconventional. For this reason, it is valuable to examine the claims that have been presented for their concordance with existing and established scientific knowledge. What controversial assertions have been made?

The four components of the argument presented include the cytogenetic analysis along with the three complementary pieces of information required to derive a comparison to the results of that analysis: calculation of the ambient concentration of Xe-133, *χ*, further calculation of the concentration of xenon in the tissue of a human being, *c*, and finally the application of shot noise statistics to determine the power dissipated in tissue due to radioactive decay (*p*_*SN*_) and the reference dose *D*_*ref*_. Regarding the concentrations of xenon-133 in the atmosphere and in human tissue, although the values utilized in this report are not accurately known with high confidence, the claims are entirely conventional. While the BDC results for the source term and greatest exposure are a few times higher than the conventional belief as expressed by the Kemeny Commission report (22 MCi vs. 2.4–11 MCi and 2.1 mSv vs. 0.7 mSv), the atmospheric modeling was performed carefully following established methods^75^, and there seems to be no basis upon which to question atmospheric concentrations of xenon-133 on the order of 1 μCi/l persisting for multiple hours. Furthermore, the basic claims that the gas passes easily from the atmosphere into the bloodstream and accumulates preferentially in fatty tissue, from which it is slowly released on a time scale of hours, are firmly established experimentally.

Regarding the shot noise dissipated power and the reference dose, on the other hand, certainly a claim has been made contrary in at least some respects to established belief in the field of health physics. If Eq. (9) is correct, for instance, then it follows that no radiation weighting factor w_R_ can be defined for exposure to an internally incorporated beta-emitting radionuclide^76^. The statement certainly contradicts the conventional belief that w_R_ = 1 for exposure to beta radiation^77^. Nevertheless, the expression of Eq. (6) for the shot noise dissipated power is precisely analogous to the Schottky result for the shot noise current in an electrical circuit, a century-old finding well-known within the electrical engineering and applied mathematics communities^78^. While undoubtedly new information to workers in health physics and radiobiology, the expression for the shot noise dissipated power therefore possesses a solid foundation and must be considered firmly established. Derivation of the reference dose expression of Eq. (9) additionally requires only the redefinition of the energy imparted to tissue to properly account for the temporal character of energy deposition. The adjustment is fully justified simply by the elementary statement that energy is the time integral of dissipated power.

For this reason, while the results of the reference dose calculations given in Table 4 may rightly be considered surprising, the theoretical foundation supporting the calculations must be considered sound and well-established. Beyond the theory, however, the expression of Eq. (9) for the shot noise reference dose has in fact been verified experimentally in a relevant model system^76,79^. Future investigation is urgently needed and may offer additional support. Since both isotopes are beta-emitters, for instance, existing work examining tritium^80,81^ or the medical administration of I-131^82–84^ may additionally validate the presence of shot noise in a radiobiological system in vivo.

It remains to consider the cytogenetic analysis, which was performed by qualified experts, employed the accepted FISH method examining stable chromosome aberrations, and followed applicable IAEA guidelines^85^. While the results of cytogenetic analysis of a small number of persons should not be fully embraced without appropriate skepticism, it would at a minimum be possible for outside experts to review the authors’ conclusions on the basis of their published observations. A more vigorous approach, however, would be to undertake a rigorous, transparent, and comprehensive program of cytogenetic testing in the present day. It is only forty years since the 1979 accident at Three Mile Island. The Russian experience with the Altai population—where the time lag between exposure and cytogenetic testing was forty-three years—confirms the feasibility of the proposal. Many of the individuals affected by the meltdown at Three Mile Island in Pennsylvania may still be alive, could be identified, and might be willing to cooperate with investigation, including cytogenetic testing and verification of their location on 28–29 March 1979. Persons who did not exhibit obvious signs of radiation sickness at the time of the accident could easily still have received reference doses exceeding the detection threshold.

A short statement about error analysis is also appropriate. The reasonable numerical agreement between the results of cytogenetic testing (600–900 mSv) and theory (820–1,700 mSv) is a statement of best knowledge, but it is perhaps better understood as the result of a model rather than a statement of fact. In the model, the BDC results for Xe-133 emission rates and atmospheric dispersion are assumed to be strictly correct, and the affected individuals are assumed to have been in place, out of doors, at the single location identified by BDC as suffering the greatest gamma exposure (Tract 74) at the time of the accident. Whether these two assumptions are correct dominates all other contributions to the overall uncertainty, including for instance uncertainties involving the free radical lifetime (which could be either more or less than 1 ns), the interaction volume (which might be judged larger if the number of electron tracks simulated were increased), the parameters describing the biokinetics of inhaled xenon, the effect of shielding (if any), and even the dose rate factor of 2–3 × applied to the results of cytogenetic analysis. A strict quantitative judgment is therefore not really supportable. Instead, a justifiable conclusion is that a discrepancy of three orders of magnitude (that is, the 2.1 mSv gamma exposure, based upon physical dosimetry, versus 600—900 mSv by biodosimetry) has been reduced to zero orders of magnitude (a factor of less than two). Although it remains to investigate many specific details, shot noise resolves what otherwise appears to be an insurmountable paradox.

In closing, there is actually one location where the ambient concentration of Xe-133 due to the most intense release was directly measured. Scientists working for the New York State Department of Health in Albany reported these results in *Science* magazine^48^. Using cryogenic separation and beta spectroscopy, the researchers measured Xe-133 concentrations of 3.1–3.9 pCi/l in air samples taken between 15:00 h on 30 March and 01:45 h 31 March 1979. They also deployed a semiconductor detector to observe the 81 keV Xe-133 gamma ray line in ambient air in the laboratory, from which results of 1.4 and 1.1 pCi/l over sampling periods from 12:30 29 March to 15:00 30 March and 15:30 30 March to 08:30 2 April were obtained. Using the ambient activity values, Wahlen and colleagues calculated whole-body doses to individuals in the Albany area of 40 nSv due to the passing cloud of Xe-133.

The data do not provide a precise means to determine the onset of exposure in Albany in 1979, but by way of a reasonable comparison one might consider exposure to a dilute cloud of Xe-133 at a concentration of 1 pCi/l persisting for 24 h. Following the calculational framework presented in this report the shot noise reference dose in this case comes to around 2–3 mSv, which is about the same as the BDC result for the greatest gamma ray dose encountered in the vicinity of the Three Mile Island facility at the time of the accident. The discrepancy between the calculated whole-body gamma ray dose and the shot noise reference dose representing the true biological impact to those persons living in Albany, NY, in 1979 is of the order of fifty thousand times.

## Conclusion

While the conventional belief is that no one was harmed by exposure to ionizing radiation due to the 1979 accident at the Three Mile Island nuclear power plant, actually the most comprehensive epidemiological evidence addressing the question is equivocal. Researchers have found that the population near the facility does appear, by comparison to control (unexposed) populations, to have suffered health decrements including cancer, cardiac disease, and early mortality. Because the whole-body gamma ray doses are deemed too small to have been causative of the observed medical impacts, however, researchers concluded that exposure to ionizing radiation cannot have been responsible for the health impacts observed.

The fundamental contribution made by this report is the presentation of evidence pointing out a serious logical flaw in the exclusionary reasoning employed: it relies upon the health physics body of knowledge, which is incomplete in an important and fundamental respect. While dose measures solely the energy dissipated in tissue, without regard for its temporal distribution, the chemical impact of dilute contamination with the beta-emitting noble gas Xe-133 results precisely from the instantaneous temporal distribution of ionization events (and not at all from the dose, which is indeed negligible). The definition of the energy imparted to tissue, ε, must be expanded to properly represent the very large departures from chemical equilibrium caused by beta emission due to an internally-incorporated radionuclide. With this single, simple, and well-justified modification to the definition of a single microdosimetric parameter, reference doses to the most-exposed population near Three Mile Island in the range from 820 to 1,700 mSv are obtained. The results are of similar magnitude (larger by less than a factor of two) to the results of cytogenetic analysis showing exposures in the range of 600–900 mSv.

The information presented therefore represents a paradigm shift^86^ in the field of health physics. If a gamma ray dose not greater than 2 mSv were indeed the greatest exposure suffered by any individual near Three Mile Island at the time of the accident, then it would in fact be impossible to engage usefully with the results of cytogenetic analysis indicating exposures of nearly 1,000 mSv. It is not possible, in the real world, to chase down every lead, to engage with every experimental finding, or to reconcile every assertion. With the result of Eq. (9) for the shot noise reference dose, however, the results of cytogenetic analysis—possibly as well as the epidemiological findings—are suddenly easily explainable. It is merely a matter of detail to resolve a discrepancy of about a factor of two. It follows that the true history of the Three Mile Island accident and its impacts upon the health of the surrounding population may remain to be written.

The work has significant implications at many levels and deserves widespread attention, not limited to re-evaluation of existing knowledge or past epidemiological investigations. It does appear that a substantial revision to the body of knowledge in the field of health physics is required to bring it into concordance with a well-established scientific result, published a century ago, deriving from the discrete nature of the electron. Because the health physics body of knowledge is incomplete as regards the biological action of dilute concentrations of internally-incorporated beta-emitting radionuclides, existing work in the area of reactor accident consequence analysis^87^ is not constructed upon a firm scientific foundation, and regulations governing batch releases from operating nuclear reactors^88^ are not protective to the public as intended. Moreover, if the conventional understanding that no one was harmed by the Three Mile Island accident is incorrect—as it happens, due to the failure to apply elementary calculus—then society-wide arguments about the safety, utility, desirability, and necessity of nuclear power have been badly misinformed for more than a generation. Further investigation is urgently needed, including hopefully the institution of a comprehensive program of cytogenetic testing focused upon those persons still alive who suffered exposure to the plume of Xe-133 released during the accident at Three Mile Island forty years ago. The subject population would be restricted to willing participants who can be positively identified and whose locations on 28–29 March 1979 are known with certainty.

### Statement on use of experimental animals or human subjects

The author declares that he has performed no experimental work involving animal subjects or human participants in the course of his research.
